# Supplementary material for: Oxidized sulfur-rich arc magmas formed porphyry Cu deposits by 1.88 Ga
Source: Nat Commun. 2021 Apr 13;12:2189. doi: 10.1038/s41467-021-22349-z (PMC8044198; doi:10.1038/s41467-021-22349-z)
Supplement: Supplementary file 1 — Supplementary Information [file 41467_2021_22349_MOESM1_ESM.pdf]

Supplementary Information

Oxidized sulfur-rich arc magmas formed porphyry Cu deposits by 1.88 Ga

Xuyang Meng[1, *], Jackie M. Kleinsasser[2], Jeremy P. Richards[1, †], Simon R. Tapster[3], Pedro J. Jugo[1], Adam C. Simon[2], Daniel J. Kontak[1], Laurence Robb[4, 5], Grant M. Bybee[6], Jeffrey H. Marsh[1], and Richard A. Stern[7]

[1] Mineral Exploration Research Centre, Harquail School of Earth Sciences, Laurentian University, Sudbury, Ontario P3E 2C6, Canada

[2] Department of Earth and Environmental Sciences, University of Michigan, Ann Arbor, Michigan 48109, USA

[3] Geochronology and Tracers Facility, British Geological Survey, Keyworth, Nottingham NG12 5GG, UK

[4] Department of Earth Sciences, University of Oxford, Oxford OX1 3AN, UK

[5] DSI-NRF Centre of Excellence, University of Johannesburg, Johannesburg 2001, South Africa

[6] School of Geosciences, University of Witwatersrand, Johannesburg 2001, South Africa

[7] Canadian Centre for Isotopic Microanalysis, University of Alberta, Edmonton, Alberta T6G 2E3, Canada

* Corresponding author, email: xmeng1@laurentian.ca

† Deceased, June 7, 2019

Supplementary Note 1: the Haib porphyry Cu deposit

The Haib porphyry Cu deposit is located in the western Richtersveld Magmatic Arc of southern Namibia, where the arc assemblage mainly comprises the greenschist facies Orange River Volcanic Group and underlying younger Vioolsdrif Plutonic Suite (Fig. 1). In Haib, the Vioolsdrif Plutonic Suite is remarkably undeformed compared to the localized intense deformation of the Orange River Volcanic Group along the NW-trending shear zones (Fig. 1), which may reflect rheological contrasts between strong crystalline plutonic rocks and the weaker phyllosilicate-rich volcanic rocks. General lack of deformation in the volcanic rocks within Haib and near contacts with the granodiorite intrusions may indicate local induration by contact metamorphism prior to the regional deformation. Metamorphism to greenschist grade occurs during the ~1.1 Ga Namaqua Orogeny [1] and has variably affected the rocks in Haib.

Lithology

The rocks in the Haib deposit include pre-mineralization plagioclase-phyric andesite porphyry and interleaved rhyolitic tuff, syn-mineralization granodiorite porphyry and leucocratic granodiorite porphyry, and post-mineralization aplite dikes (Supplementary Figs. 1–3). The rocks in the regional batholith (i.e., Vioolsdrif Plutonic Suite) mainly include unmineralized equigranular granodiorite, diorite, and aplite (Supplementary Figs. 1, 2). The petrographic descriptions for the samples studied are listed in Supplementary Data 1.

The granodiorite porphyry locally contains xenoliths of plagioclase-phyric andesite porphyry and is intruded with a sharp contact by leucocratic granodiorite porphyry dikes, as observed in drill core (Supplementary Fig. 2c–e). Granodiorite in the

batholith locally contains quartz monzonite enclaves (Supplementary Fig. 2f). Aplite dikes cut the granodiorite porphyry and intrude equigranular granodiorite of the batholith (Supplementary Fig. 2h and i). The dike is poorly mineralized and is crosscut by late quartz-ankerite veins, which is interpreted to have formed later than the main porphyry Cu-mineralization stage.

Note that the plagioclase-phyric andesite porphyry, granodiorite porphyry, and leucocratic granodiorite porphyry are formerly referred to as feldspar porphyry, quartz-feldspar porphyry, and quartz-feldspar porphyry II in refs. [2, 3], and reports from the Deep Resources, Inc. A phase of quartz biotite porphyry is also identified, but its geochronology and lithogeochemical composition are comparable to the granodiorite porphyry [3]. We therefore argue that petrographic differences may reflect various degrees of alteration. A mineralized dioritic feldspar porphyry dike containing granodiorite porphyry xenolith is identified (Supplementary Fig. 1b), which is interpreted to be a breccia [3] and is not studied here.

Alteration

Alteration at Haib has been described in detail in refs. [2, 3], and the noted alteration assemblages are comparable to Phanerozoic porphyry Cu systems [4, 5], which include early potassic alteration with local overprinting by albite, chlorite, and sericite alteration types (Supplementary Fig. 1c). Pervasive biotite and minor K-feldspar alteration are commonly associated with epidote, anhydrite, muscovite, and minor tourmaline, titanite, and rutile (Supplementary Fig. 4a–f). Epidote is rarely associated with potassic alteration and mineralization in typical Phanerozoic porphyry Cu systems, and its presence in Haib may reflect the hydrothermal fluid being more

calcic. Local K-feldspar selvages are seen around veins, and most of this alteration has associated minor molybdenite mineralization.

Albite alteration is rare and was only identified in the granodiorite porphyry at a depth of ~780 m in drill hole TCDH-10 (Supplementary Fig. 1c). It is characterized by replacement of K-feldspar with albite and is associated with low-Cu grades.

Chlorite alteration is mainly observed in the plagioclase-phyric andesite porphyry (Supplementary Fig. 2a) and part of the granodiorite porphyry intrusion (Supplementary Fig. 2h). Two stages of epidote-chlorite alteration are identified in the plagioclase-phyric andesite porphyry (Supplementary Figs. 2a, 4g), which may be associated with thermal metamorphism of this unit during intrusion of the granodiorite porphyry and later hydrothermal alteration [3].

Sericite alteration is locally seen in drill core and mainly replaces plagioclase and biotite (Supplementary Fig. 4h). At the property surface, which is considered as the shallow levels of the system, the potassic alteration is locally overprinted by sericite alteration whereby sericite completely replaces plagioclase.

Regional metamorphism during Namaqua Orogeny [1] is mainly characterized by the presence of epidote, sericite, and minor chlorite, and has variably affected all of the rock types in the area.

Veining and mineralization

The deposit contains disseminated and veinlet chalcopyrite ± pyrite mineralization mainly associated with biotite ± K-feldspar ± anhydrite ± titanite ± rutile ± epidote alteration. Magnetite is exceptionally rare. The early dark micaceous (EDM)

alteration selvages contain biotite ± epidote ± muscovite ± K-feldspar ± albite ± titanite ± rutile which are crosscut by sinuous and milky quartz A veins (Supplementary Fig. 4d, e).

The sinuous quartz A veins, with granular textures, contain relatively minor amounts of sulfide minerals, mainly chalcopyrite, and are in equilibrium with potassic alteration in the wall rock, but also locally have narrow K-feldspar alteration halos (Supplementary Fig. 4f).

Quartz ± molybdenite ± chalcopyrite ± pyrite B veins are rarely found and pyritic D veins are minor. The D veins with chlorite-sericite (in the plagioclase-phyric andesite porphyry) or feldspar-destructive sericite (in the granodiorite porphyry) alteration halos follow the main stage of Cu mineralization (Supplementary Fig. 4g, h). The lack of widespread sericite alteration and B and D veins may reflect the relatively deeper level of the system. The localized quartz-tourmaline veins seem to have not introduced significant sulfide mineralization (Supplementary Fig. 4j). Carbonate veins crosscut the quartz-tourmaline veins and are similarly barren.

Supplementary Note 2: apatite crystallized prior to volatile saturation

Thermodynamic models in ref. [6] predict that apatite halogen compositions evolve along different trajectories during volatile-undersaturated and water-saturated crystallization. (1) During volatile-undersaturated crystallization, the X_F/X_{OH} ratio decreases while the X_{Cl}/X_{OH} ratio (X_{Cl}, X_F, and X_{OH} are mole fractions of Cl, F, and OH in apatite) varies depending on the exact values of the partition coefficients for F, Cl, and OH between apatite and melt. (2) Because the partition coefficient for Cl in

the fluid is much higher than for F [7, 8], during water-saturated crystallization the apatite X_{Cl}/X_{OH} ratio sharply decreases with constant or slightly increasing X_F/X_{OH} ratio.

To evaluate the relative timing of crystallization of titanite/zircon-hosted apatite to volatile saturation in parent melt for Haib, we plot apatite halogen compositions in Supplementary Fig. 9. We restrict the plotting to analyses containing Cl content above the detection limit (190 ppm), and these analyses are mainly for samples HB-30 (diorite) and HB-51 (leucocratic granodiorite porphyry). One single analysis is also available for granodiorite (sample HB-34) but cannot form a trend in itself, and is therefore not included. The c-axis of most of the apatite grains are perpendicular to the electron beam, so that beam damages are interpreted to have been minimized (see the Methods section in the main text).

The apatite X_F/X_{OH} ratio decreases with decreasing X_{Cl}/X_{OH} ratio, which is consistent with the modeling result for apatite crystallized in a volatile-undersaturated environment in ref. [6], suggesting that the apatites crystallized earlier than magmatic degassing. This is also consistent with the fact that apatite from individual samples yielded limited compositional variability (Supplementary Data 9) and lacks zoning (Supplementary Fig. 9).

Supplementary Note 3: P-T correction of magmatic fO_2 from apatite S μ-XANES data

In silicate glasses, it has been demonstrated that a decrease in temperature of 100 °C and a decrease in pressure of 300 MPa may result in an approximate deviation of ΔFMQ + 0.5 and -0.2, respectively [9, 10]. Given that apatite may crystallize as a near-liquidus phase, we use the *model* apatite saturation temperature (AST) [11] to constrain the temperature at which the majority of the zircon-hosted apatite crystallized. The P-T-corrected magmatic fO_2 for the plutonic rocks (n = 4, AST = 914 ± 4 °C for granodiorite and diorite emplaced at ~200–300 MPa [2]; samples HB-18, HB-30, HB-32, and HB-51) yielded a narrow range of ΔFMQ +1.22 to +1.25 (1σ, 1.24 ± 0.01 on average). For subaerial volcanism, which presumably erupted at near-atmospheric pressure, results yielded an AST of 868 ± 1 °C (n = 2), the average $\log fO_2$ values for samples HB-24 (plagioclase-phyric andesite porphyry) and HB-28 (rhyolitic tuff) are ΔFMQ +1.37 ± 0.02 (1σ).

Supplementary Note 4: sources of geochemical datasets compiled for Fig. 5.

1. Data of apatite S concentration compiled from the GEOROC database (Geochemistry of Rocks of the Oceans and Continents; http://georoc.mpch-mainz.gwdg.de/georoc/) and refs. [12, 13, 14, 15, 16].
2. Data of melt S inclusion for arc magmas are compiled from GEOROC. Melt inclusion data for rocks associated with Phanerozoic porphyry Cu systems are from refs. [17, 18, 19].

Supplementary Figures

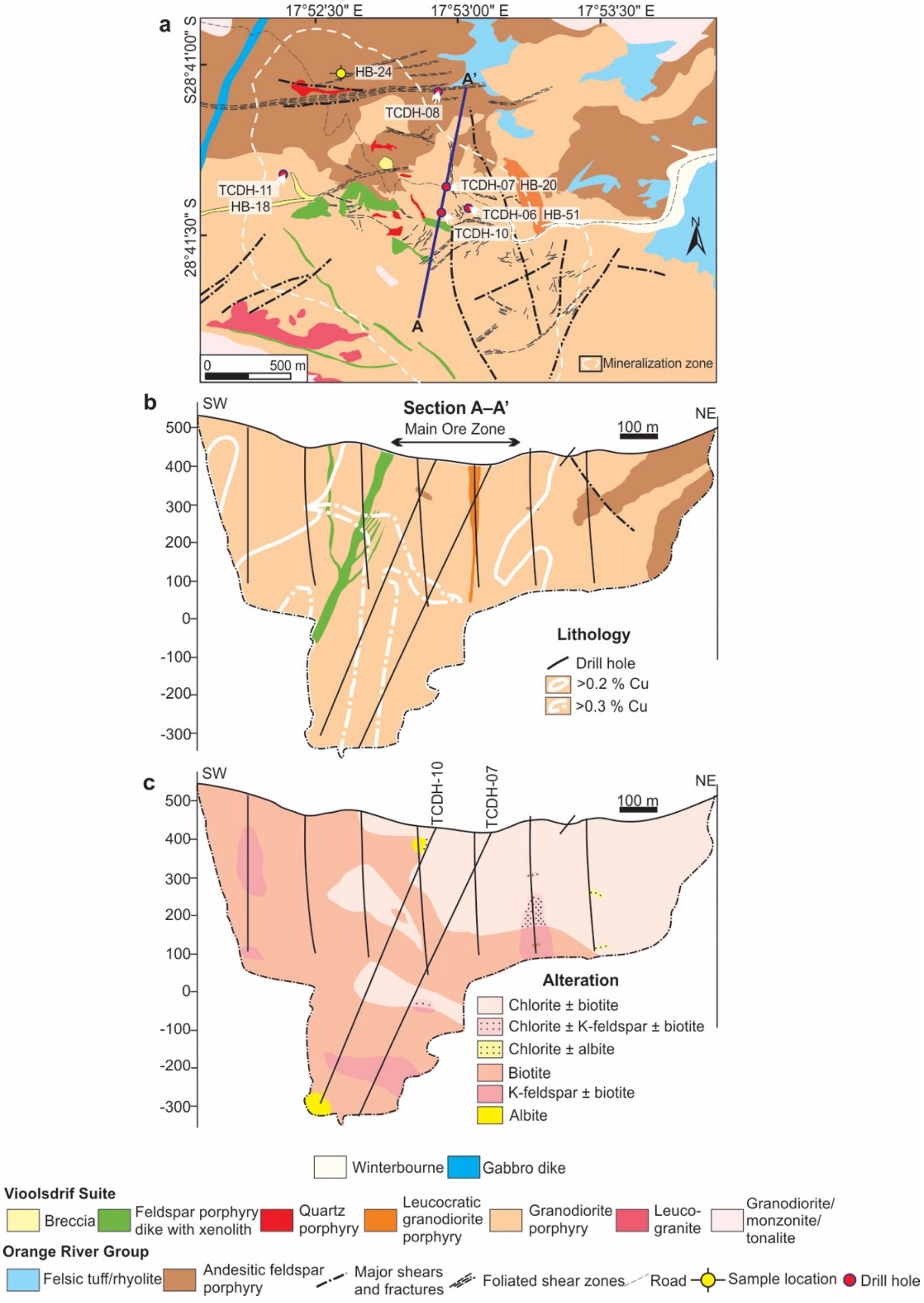

Supplementary Fig. 1 | Geological map and cross section of the Haib deposit. a, Geological map of the Haib deposit, showing lithological units and their geometry, modified from ref. [20]. The defined mineralization zone is made by Deep South Resources, Inc.. **b,** cross section of lithology and Cu-grade contours, **c,** cross section of alteration. Location of the section shown in Supplementary Fig. 1a. The cross sections were modified from the Deep South Resources, Inc. (unpublished report).

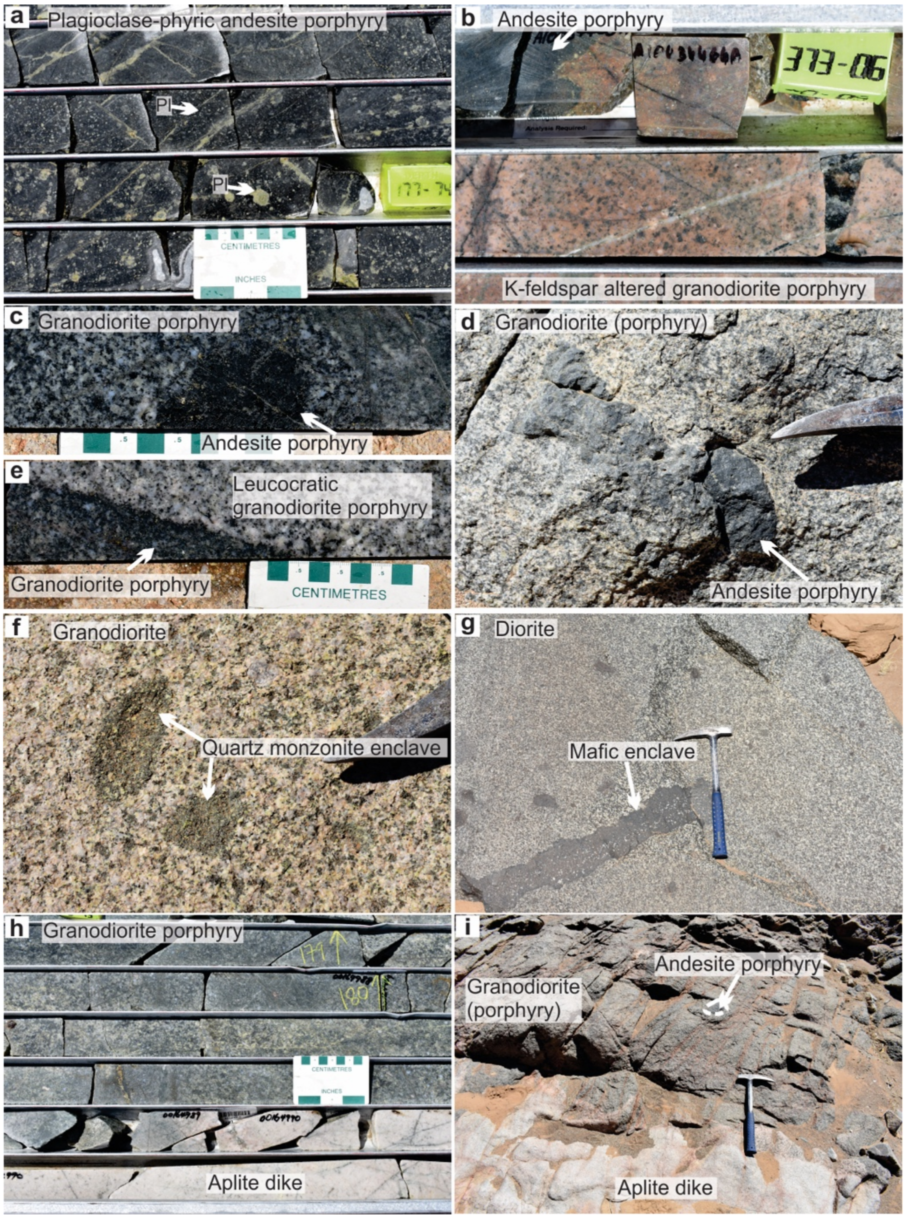

Supplementary Fig. 2 | Drill core and field photographs of igneous rocks in the Haib area. a, Plagioclase-phyric andesite porphyry with epidote-altered plagioclase phenocrysts (TCDH-08, 173.7–180.1 m). **b,** Andesite porphyry intruded by granodiorite porphyry with quartz phenocrysts (K-feldspar altered and hematite stained; TCDH-11, 373 m). **c,** Granodiorite porphyry with xenolith of andesite porphyry (TCDH-06, 741–741.2 m). **d,** Plagioclase-phyric andesite porphyry xenolith in granodiorite porphyry (UTM 33J 0781257 6823338, 452 m, WGS 84 datum). **e,** Leucocratic granodiorite porphyry dike in granodiorite porphyry (TCDH-06, 742–742.3 m). **f,** Granodiorite in batholith and its quartz monzonite enclave (UTM 33J 0783664 6827781, 369 m). **g,** Diorite in the batholith with xenolith of mafic to ultramafic composition (UTM 33J 0785311 6827730, 232 m). **h,** Aplite dike cutting the granodiorite porphyry (chlorite-altered; TCDH-07, 182–183 m). **i,** Aplite dike cutting the granodiorite (porphyry) with plagioclase-phyric andesite porphyry xenolith (UTM 33J 0781257 6823338, 452 m). Abbreviations: Pl = plagioclase. Drill hole collar locations can be seen in Supplementary Data 1.

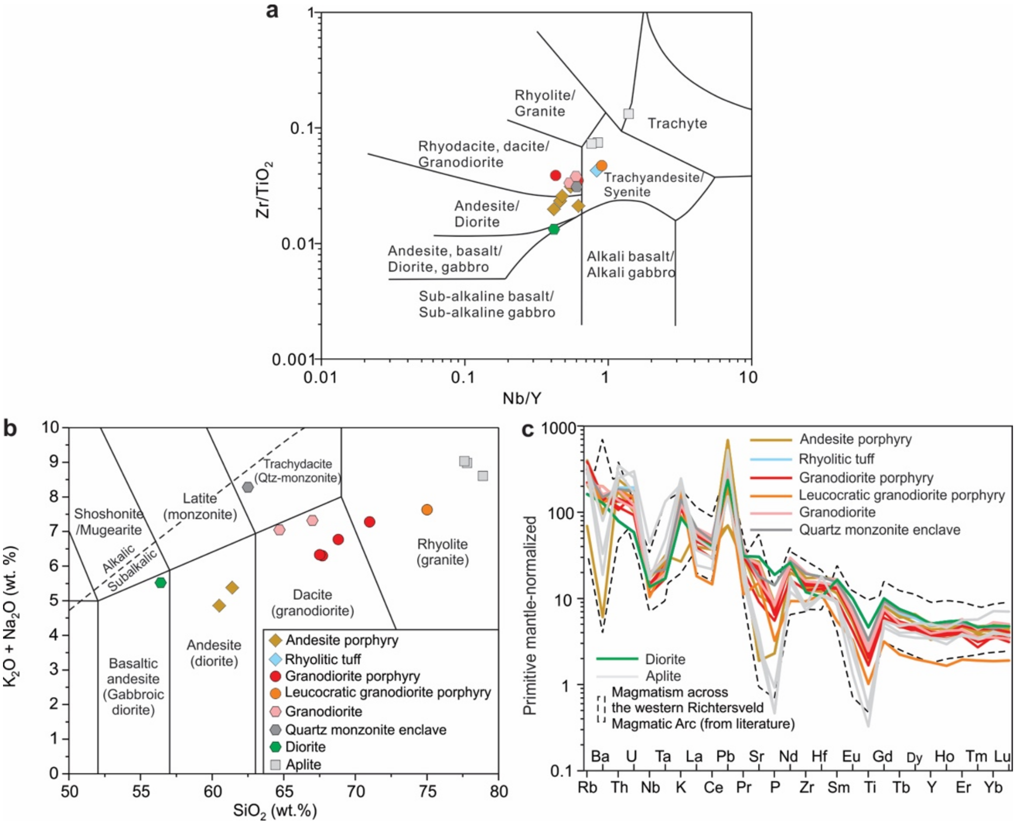

Supplementary Fig. 3 | Summary of whole-rock geochemistry of the igneous rocks from Haib and the western Richtersveld Magmatic Arc. a, Zr/TiO_2 versus Nb/Y discrimination diagram [21] for the major rock types from Haib. **b,** Total alkali-silica diagram [22] for the major rock types from Haib (samples with LOI > 2 wt. % were excluded). The alkali/subalkalic boundary line is from ref. [23]. **c,** Primitive mantle-normalized trace-element spider diagram. The normalization values for (c) are from ref. [24]. Data for the igneous rocks from the western Richtersveld Magmatic Arc were compiled in ref. [25].

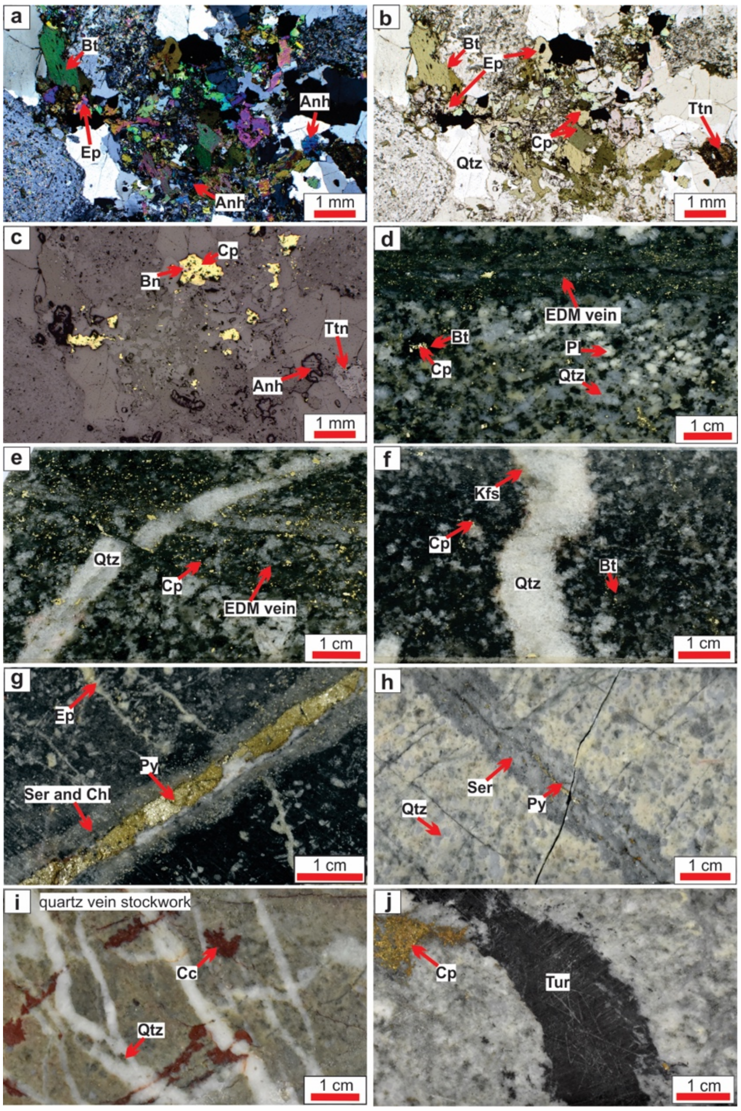

Supplementary Fig. 4 | Photographs and photomicrographs of alteration and vein types. a–c, Biotite ± epidote ± anhydrite ± titanite alteration associated with chalcopyrite ± bornite mineralization in granodiorite porphyry (sample HB-53; TCDH-06, ~749.8 m); **a,** thin section cross-polarized, **b,** plane-polarized, and **c,** reflected light. **d,** Granodiorite porphyry with early dark micaceous (EDM) alteration selvage and disseminated chalcopyrite mineralization (sample HB-43, TCDH-10, ~365 m). **e,** EDM vein crosscut by A-type quartz vein in the granodiorite porphyry (TCDH-06, ~755 m). The A-type quartz vein is offset by a fracture. **f,** Sigmoidal A-type quartz vein with minimal sulfide mineralization and minor K-feldspar along margin in biotite-altered granodiorite porphyry (TCDH-06, below 755 m). **g,** Pyritic D-vein with sericite-chlorite alteration halo in the epidote-altered plagioclase-phyric andesite porphyry (TCDH-08, ~175 m). **h,** Pyritic D-vein with sericite alteration halo in granodiorite porphyry (TCDH-11, ~377 m). **i,** Quartz vein stockwork in granodiorite porphyry (TCDH-06, 762.5–762 m) with high-Cu grade (1.2–1.4 % Cu), possibly reflecting oxidation and formation of secondary chalcocite. **j,** Tourmaline vein in sericite-altered granodiorite porphyry (sample HB-49, TCDH-06, ~606 m). Abbreviations: Anh = anhydrite, Bn = bornite, Bt = biotite, Cc = chalcocite, Chl = chlorite, Cp = chalcopyrite, Ep = epidote, Kfs = K-feldspar, Py = pyrite, Qtz = quartz, Ser = sericite, Ttn = titanite, Tur = tourmaline. See drill hole collar location in Supplementary Data 1 and Supplementary Fig. 1.

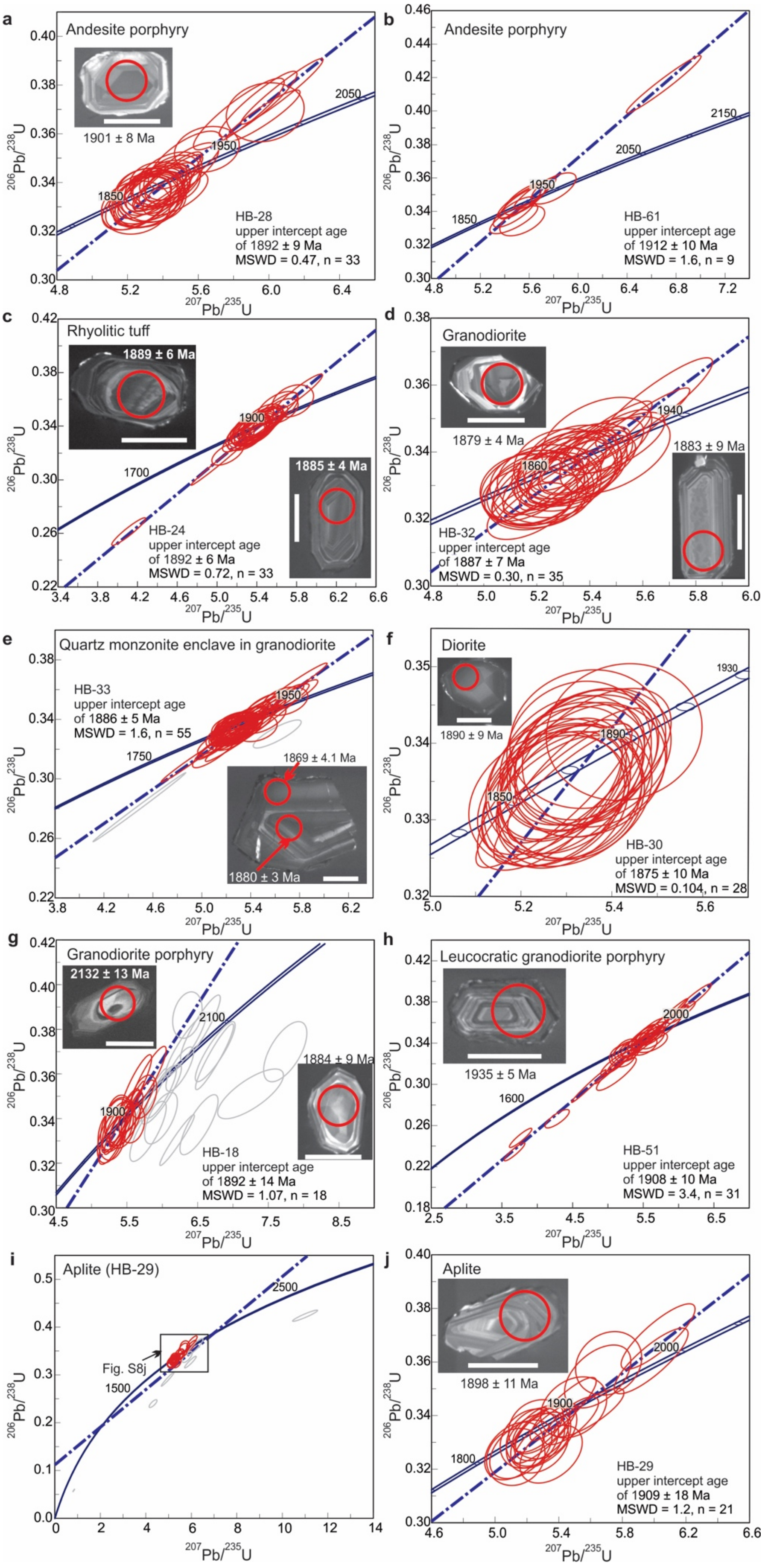
a
Andesite porphyry
1901 ± 8 Ma
HB-28
upper intercept age
of 1892 ± 9 Ma
MSWD = 0.47, n = 33
1850
1950
2050
b
Andesite porphyry
HB-61
upper intercept age
of 1912 ± 10 Ma
MSWD = 1.6, n = 9
1850
1950
2050
2150
c
Rhyolitic tuff
1889 ± 6 Ma
1885 ± 4 Ma
HB-24
upper intercept age
of 1892 ± 6 Ma
MSWD = 0.72, n = 33
1700
1900
d
Granodiorite
1879 ± 4 Ma
1883 ± 9 Ma
HB-32
upper intercept age
of 1887 ± 7 Ma
MSWD = 0.30, n = 35
1860
1940
e
Quartz monzonite enclave in granodiorite
HB-33
upper intercept age
of 1886 ± 5 Ma
MSWD = 1.6, n = 55
1869 ± 4.1 Ma
1880 ± 3 Ma
1750
1950
f
Diorite
1890 ± 9 Ma
HB-30
upper intercept age
of 1875 ± 10 Ma
MSWD = 0.104, n = 28
1850
1890
1930
g
Granodiorite porphyry
2132 ± 13 Ma
1884 ± 9 Ma
HB-18
upper intercept age
of 1892 ± 14 Ma
MSWD = 1.07, n = 18
1900
2100
h
Leucocratic granodiorite porphyry
1935 ± 5 Ma
HB-51
upper intercept age
of 1908 ± 10 Ma
MSWD = 3.4, n = 31
1600
2000
i
Aplite (HB-29)
Fig. S8j
1500
2500
j
Aplite
1898 ± 11 Ma
HB-29
upper intercept age
of 1909 ± 18 Ma
MSWD = 1.2, n = 21
1800
1900
2000
$^{206}Pb/^{238}U$
$^{207}Pb/^{235}U$

Supplementary Fig. 5 | Zircon U-Pb concordia diagrams for the major rock units from Haib; 2σ error ellipses are shown. MSWD represents the mean square of weighted deviates. For these samples, upper intercept concordia ages were calculated using Isoplot v. 4.5 to define the crystallization ages. Uranium decay constant uncertainties are not considered. **a and b**, Plagioclase-phyric andesite porphyry (samples HB-28 and HB-61). **c,** Rhyolitic tuff (sample HB-24). **d,** Granodiorite (sample HB-32). **e,** Quartz monzonite xenolith in granodiorite (sample HB-33, two discordant analyses excluded are indicated in grey ellipses). **f,** Diorite (sample HB-30). **g,** Granodiorite porphyry (sample HB-18; analyses with common lead excluded from the regression are indicated in grey error ellipses). The common lead may come from subsurface phosphate-rich inclusions, such as apatite, as demonstrated by high P content (thousands of ppm; Supplementary Data 4). **h,** Leucocratic granodiorite porphyry (sample HB-51). **i,** Aplite (sample HB-29). **j,** Aplite (sample HB-29; with six discordant analyses excluded from the upper concordia age determination as indicated in grey ellipses in Supplementary Fig. 5i). Insets are CL images of representative zircons with $^{207}Pb/^{206}Pb$ ages (2 standard errors) reported. The red circles indicate the spots for laser ablation. The white bars below the CL images are 50 µm scale bars. See Supplementary Data 1 for sample locations and descriptions.

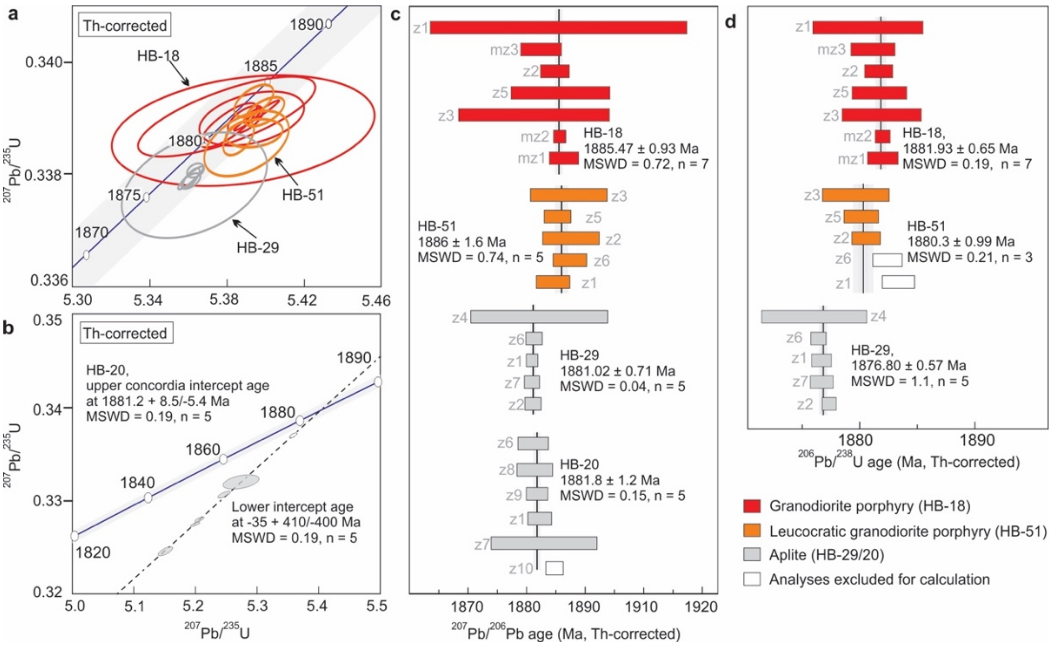

Supplementary Fig. 6 | CA-ID-TIMS zircon U-Pb dating results for representative igneous rocks from the Haib deposit. **a and b,** Wetherill concordia diagram of CA-ID-TIMS U-Pb isotope analyses. **c,** Plot of zircon $^{207}Pb/^{206}Pb$ dates for samples HB-18, HB-51, HB-29, and HB-20. **d,** Plot of zircon $^{206}Pb/^{238}U$ dates for samples HB-18, HB-51, and HB-29. The grey bands along the concordia lines in (a) and (b) indicate uranium decay constant uncertainties. Error bars and ellipses represent 2σ uncertainties for single zircon fractions.

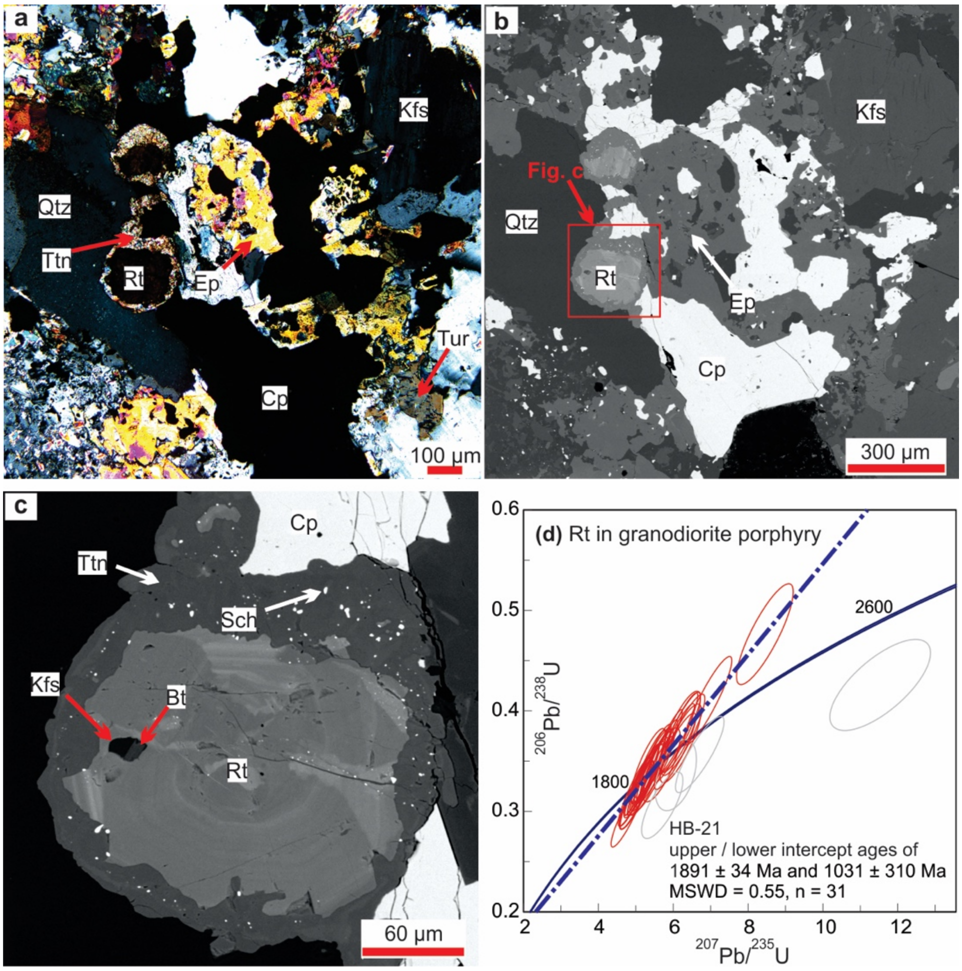

Supplementary Fig. 7 | LA-ICP-MS U-Pb dating of hydrothermal rutile intergrown with chalcopyrite. a and b, Subhedral hydrothermal rutile with sector and oscillatory zoning intergrown with epidote, K-feldspar, and chalcopyrite in the granodiorite porphyry (sample HB-21); cross-polarized light and BSE. **c,** The zoned rutile replaced by titanite in the granodiorite porphyry (sample HB-21); BSE. **d,** U-Pb concordia diagram for rutile sample HB-21; four analyses excluded from the upper intercept concordia age determination are indicated in grey ellipses. All uncertainties are reported at 2σ. MSWD represents the mean square of weighted deviates. Abbreviations: Anh = anhydrite, Bt = biotite, Cp = chalcopyrite, Ep = epidote, Kfs = K-feldspar, Rt = rutile, Sch = scheelite, Ttn = titanite. See Supplementary Data 1 for sample locations and descriptions.

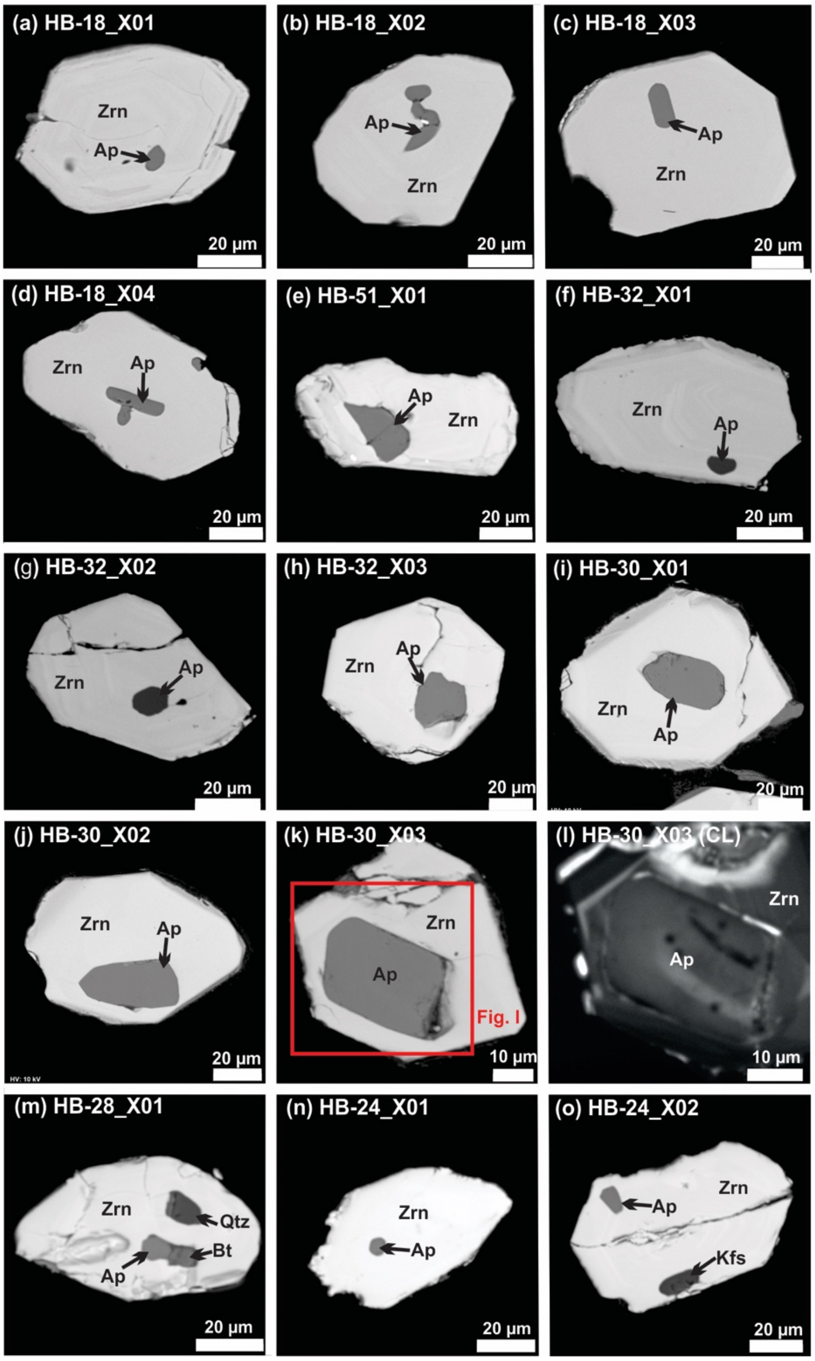

Supplementary Fig. 8 | Backscattered electron images of zircons and the apatite inclusions for µ-XANES analyses from Haib, except for image l which is in cathodoluminescence. All of these apatite inclusions are wholly enclosed in zircon and isolated from fractures. **a–d,** Sample HB-18, granodiorite porphyry. **e,** Sample HB-51, leucocratic granodiorite porphyry. **f–h,** Sample HB-32, granodiorite. **i–l,** sample HB-30, diorite. **m,** Sample HB-28, plagioclase-phyric andesite porphyry. **n and o,** Sample HB-24, rhyolitic tuff; BSE. Abbreviations: Ap = apatite, Bt = biotite, Kfs = K-feldspar, Qtz = quartz, Zrn = zircon. See Supplementary Data 1 for sample locations and descriptions.

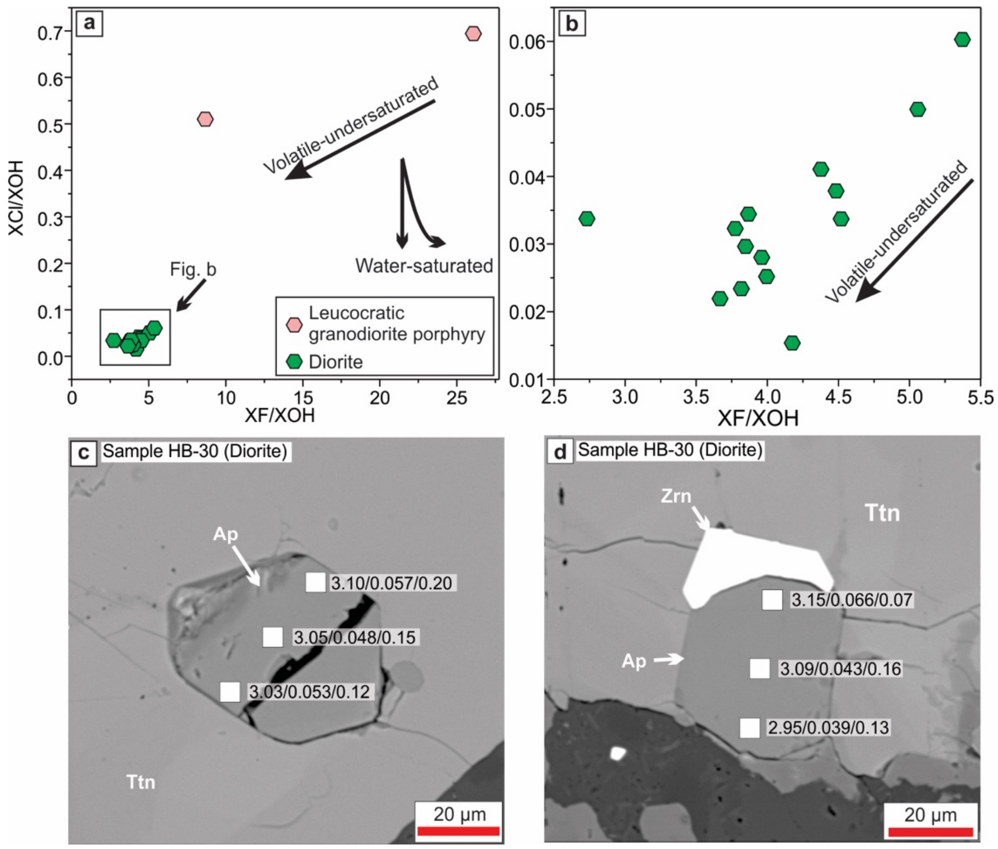

Supplementary Fig. 9 | Titanite- and zircon-hosted apatite halogen contents. a and b, Plots of X_{Cl}/X_{OH} versus X_F/X_{OH} ratios of apatite inclusions in zircon and titanite from Haib (mainly for samples HB-30 and HB-51, diorite and leucocratic granodiorite porphyry). The water-saturated and volatile-undersaturated trajectories are modified from ref. [6]. **c and d,** Backscattered electron images of apatite inclusions in titanite from sample HB-30 (diorite). Concentrations of F, Cl, and S in these two apatite inclusions are shown in wt. % (F/Cl/S). White-filled squares represent raster beam spots. No zoning has been identified. Abbreviations: Ap = apatite, Ttn = titanite, Zrn = zircon.

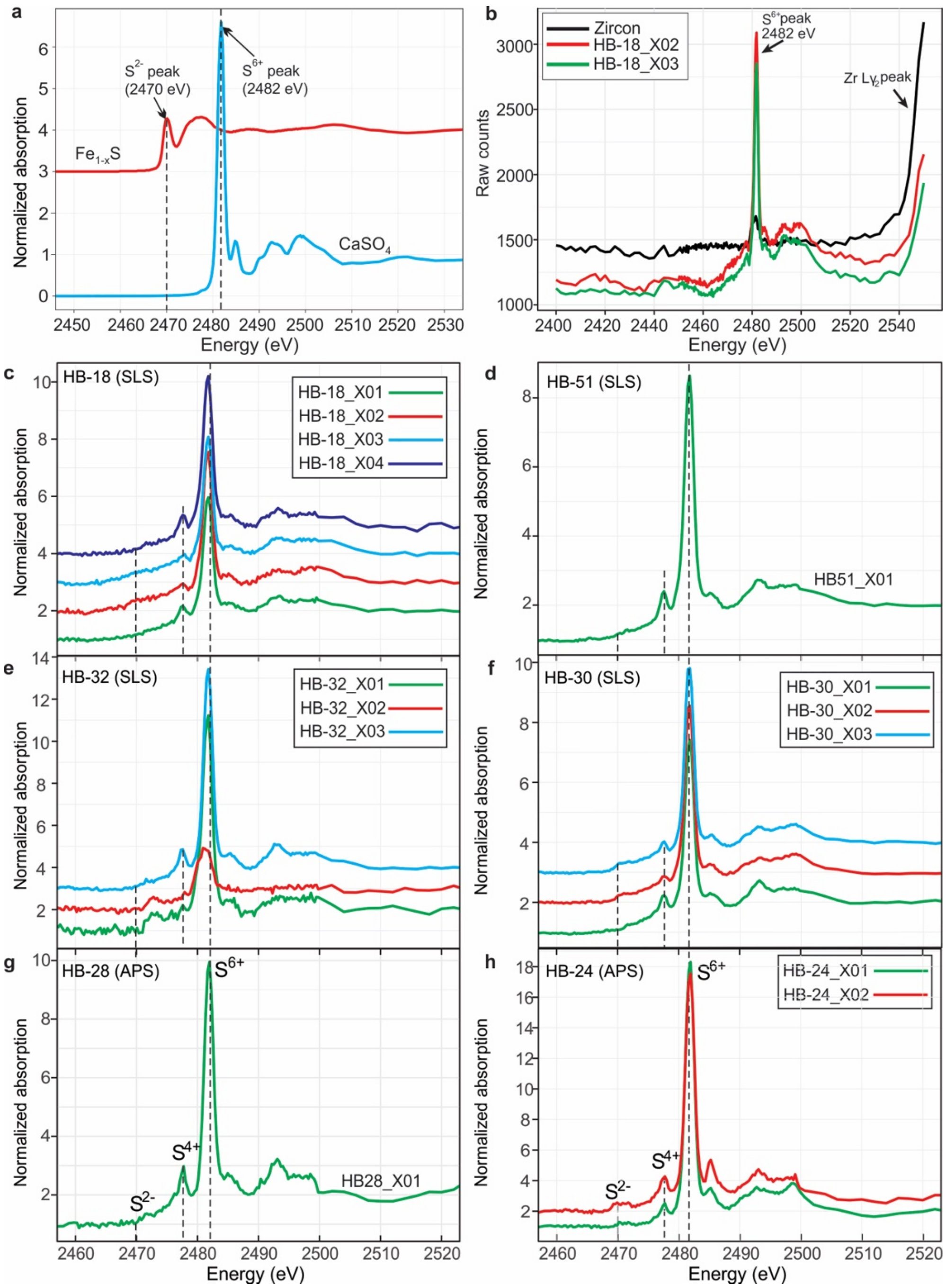

Supplementary Fig. 10 | Sulfur µ-XANES spectra of apatites and zircon from the main igneous phases in Haib, as well as reference materials. a, Reference materials pyrrhotite ($Fe_{1-x}S$) and powdered anhydrite ($CaSiO_4$). **b,** Raw counts of zircon and apatites from sample HB-18. **c,** Sample HB-18, granodiorite porphyry. **d,** Sample HB-51, leucocratic granodiorite porphyry. **e,** Sample HB-32, granodiorite. **f,** Sample HB-30, diorite. **g,** Sample HB-28, plagioclase-phyric andesite porphyry. **h,** Sample HB-24, rhyolitic tuff. The apatite spectra numbers are consistent with the apatite grain numbers in Supplementary Fig. 8. Abbreviations for laboratories: SLS = Swiss Light Source, APS = Advanced Photon Source. See Supplementary Data 1 for sample locations and descriptions.

References

1 Jacobs, J., Pisarevsky, S., Thomas, R. J. & Becker, T. The Kalahari Craton during the assembly and dispersal of Rodinia. *Precambrian Research* **160**, 142–158, doi:10.1016/j.precamres.2007.04.022 (2008).

2 Minnitt, R. C. A. Porphyry copper-molybdenum mineralization at Haib River, South West Africa/Namibia. In: *Mineral Deposits of Southern Africa* Vol II. 1567–1585 (Geological Society of South Africa, 1986).

3 Grumbley, N. L. *The Geological Evolution of the Haib Cu-Mo Porphyry, Namibia* Master thesis, Trinity College Dublin, (2015).

4 Sillitoe, R. H. Porphyry Copper Systems. *Economic Geology* **105**, 3–41 (2010).

5 Lowell, J. D. & Guilbert, J. M. Lateral and vertical alteration-mineralization zoning in porphyry ore deposits. *Economic Geology* **65**, 373–408 (1970).

6 Stock, M. J. *et al.* Tracking Volatile Behaviour in Sub-volcanic Plumbing Systems Using Apatite and Glass: Insights into Pre-eruptive Processes at Campi Flegrei, Italy. *Journal of Petrology* **59**, 2463–2492, doi:10.1093/petrology/egy020 (2018).

7 Borodulin, G. P., Chevychelov, V. Y. & Zaraysky, G. P. in *Doklady Earth Sciences.* 868–873 (Springer).

8 Webster, J. D., Tappen, C. M. & Mandeville, C. W. Partitioning behavior of chlorine and fluorine in the system apatite–melt–fluid. II: Felsic silicate systems at 200MPa. *Geochimica et Cosmochimica Acta* **73**, 559–581, doi:10.1016/j.gca.2008.10.034 (2009).

9 Nash, W. M., Smythe, D. J. & Wood, B. J. Compositional and temperature effects on sulfur speciation and solubility in silicate melts. *Earth and Planetary Science Letters* **507**, 187–198, doi:10.1016/j.epsl.2018.12.006 (2019).

10 Matjuschkin, V., Blundy, J. D. & Brooker, R. A. The effect of pressure on sulphur speciation in mid- to deep-crustal arc magmas and implications for the formation of porphyry copper deposits. *Contributions to Mineralogy and Petrology* **171**, 66, doi:10.1007/s00410-016-1274-4 (2016).

11 Piccoli, P. M. & Candela, P. A. Apatite in felsic rocks; a model for the estimation of initial halogen concentrations in the Bishop Tuff (Long Valley) and Tuolumne Intrusive Suite (Sierra Nevada Batholith) magmas. *American Journal of Science* **294**, 92–135 (1994).

12 Imai, A. Variation of Cl and SO_3 contents of microphenocrystic apatite in intermediate to silicic igneous rocks of Cenozoic Japanese island arcs:

Implications for porphyry Cu metallogenesis in the Western Pacific Island arcs. *Resource Geology* **54**, 357–372 (2004).

13 Richards, J. P. *et al.* Contrasting Tectonic Settings and Sulfur Contents of Magmas Associated with Cretaceous Porphyry Cu ± Mo ± Au and Intrusion-Related Iron Oxide Cu-Au Deposits in Northern Chile. *Economic Geology* **112**, 295–318, doi:10.2113/econgeo.112.2.295 (2017).

14 Zhu, J. J. *et al.* Elevated Magmatic Sulfur and Chlorine Contents in Ore-Forming Magmas at the Red Chris Porphyry Cu-Au Deposit, Northern British Columbia, Canada. *Economic Geology* **113**, 1047–1075, doi:10.5382/econgeo.2018.4581 (2018).

15 Imai, A. Metallogenesis of porphyry Cu deposits of the western Luzon arc, Philippines: K-Ar ages, SO_3 contents of microphenocrystic apatite and significance of intrusive rocks. *Resource Geology* **52**, 147–161 (2002).

16 Imai, A. Generation and evolution of ore fluids for porphyry Cu-Au mineralization of the Santo Tomas II (Philex) deposit, Philippines. *Resource Geology* **51**, 71–96 (2001).

17 Grondahl, C. & Zajacz, Z. Magmatic controls on the genesis of porphyry Cu–Mo–Au deposits: The Bingham Canyon example. *Earth and Planetary Science Letters* **480**, 53–65 (2017).

18 Zajacz, Z. & Halter, W. Copper transport by high temperature, sulfur-rich magmatic vapor: Evidence from silicate melt and vapor inclusions in a basaltic andesite from the Villarrica volcano (Chile). *Earth and Planetary Science Letters* **282**, 115–121 (2009).

19 Zhang, D. H. & Audétat, A. What Caused the Formation of the Giant Bingham Canyon Porphyry Cu-Mo-Au Deposit? Insights from Melt Inclusions and Magmatic Sulfides. *Economic Geology* **112**, 221–244, doi:10.2113/econgeo.112.2.221 (2017).

20 Minnitt, R. C. A. The geological setting of porphyry-type copper mineralization in the Haib River area, South West Africa. (eds). University of the Witwatersrand (1979).

21 Winchester, J. A. & Floyd, P. A. Geochemical discrimination of different magma series and their differentiation products using immobile elements. *Chemical Geology* **20**, 325–343, doi:10.1016/0009-2541(77)90057-2 (1977).

22 Le Maitre, R. W. A classification of igneous rocks and glossary of terms. *Recommendations of the international union of geological sciences subcommission on the systematics of igneous rocks* **193** (1989).
23 Irvine, T. N. J. & Baragar, W. R. A. A guide to the chemical classification of the common volcanic rocks. *Canadian journal of earth sciences* **8**, 523–548 (1971).
24 Sun, S. S. & McDonough, W. F. Chemical and isotopic systematics of oceanic basalts: implications for mantle composition and processes. *Geological Society, London, Special Publications* **42**, 313–345 (1989).
25 Macey, P. H. *et al.* Origin and evolution of the ~1.9 Ga Richtersveld Magmatic Arc, SW Africa. *Precambrian Research* **292**, 417–451, doi:10.1016/j.precamres.2017.01.013 (2017).
