# Supplementary material for: Oxidized sulfur-rich arc magmas formed porphyry Cu deposits by 1.88 Ga
Source: Nat Commun. 2021 Apr 13;12:2189. doi: 10.1038/s41467-021-22349-z (PMC8044198; doi:10.1038/s41467-021-22349-z)
Supplement: Supplementary file 3 — Description of Additional Supplementary Files [file 41467_2021_22349_MOESM3_ESM.pdf]

Description of Additional Supplementary Files

File name: Supplementary Data 1
Description: Locations and descriptions of representative igneous rocks from the Haib area.

File name: Supplementary Data 2
Description: Whole-rock geochemistry for representative igneous rocks from the Haib area.

File name: Supplementary Data 3
Description: Instrumental parameters for LA-ICP-MS U-Pb-Lu-Hf isotope and trace element analyses.

File name: Supplementary Data 4
Description: Split stream LA-ICP-MS zircon U-Pb isotope and trace element analyses for representative igneous rocks from the Haib area.

File name: Supplementary Data 5
Description: CA-ID-TIMS zircon U-Pb isotope analyses for representative intrusive rocks in the Haib area.

File name: Supplementary Data 6
Description: LA-ICP-MS U-Pb isotope analyses of hydrothermal rutile associated with chalcopyrite mineralization in the Haib deposit.

File name: Supplementary Data 7
Description: LA-ICP-MS zircon Lu-Hf isotopes for representative igneous rocks from the Haib area.

File name: Supplementary Data 8
Description: SIMS zircon O isotopes for representative igneous rocks from the Haib area.

File name: Supplementary Data 9
Description: Electron microprobe analyses of zircon/titanite-hosted apatite crystals from the representative igneous rocks from the Haib area.
